# Reducing maternal mortality in sub–Saharan Africa: the role of ethical consumerism

**DOI:** 10.7189/jogh.07.010309

**Published:** 2017-06

**Authors:** Dileep Wijeratne, Andrew David Weeks

**Affiliations:** 1 Leeds Teaching Hospitals NHS Trust, Leeds, UK; 2 Sanyu Research Unit, University of Liverpool, Liverpool, UK

Between 1990 and 2015, the maternal mortality ratio for sub–Saharan Africa as a whole fell by roughly 45 percent [1]. Unfortunately, this decline has not been uniformly distributed across the region. A number of countries have shown little or no progress and continue to experience mortality rates that rank among the highest in the world. In countries such as Angola, Liberia, Sierra Leone, Chad, Somalia and the Democratic Republic of Congo (DRC) a significant impediment to progress has been the decimation of health infrastructure by protracted regional and civil armed conflicts. During times of conflict, there is also increasing evidence of violence being directed specifically against pregnant women [2]. The 1990s saw the emergence of warfare in Africa as a means of accumulating wealth and power [3]. In–depth analyses by the United Nations and others have identified competition among various groups for the illegal expropriation and sale of the region’s vast natural resources, as both an underlying cause of conflict and also as a catalyst for ongoing conflict [4]. It has been estimated that Maternal Mortality rates are at least 30% higher in sub–Saharan African countries that have experienced recent conflict than in those which are conflict free [5]. Addressing the underlying issues relating to the protracted cycles of conflict is therefore an essential precursor to developing an environment in which interventions to address maternal mortality, or indeed any other health issue, can be implemented.

The majority of the natural resources illegally expropriated in sub–Saharan conflict areas are eventually consumed in high–income nations, such as the UK. In a number of regions, this has inadvertently provided financial capital to various armed groups [4]. For example, chronic low–level warfare and societal instability in Chad is thought to be strongly related to competition for control of the country’s oil reserves. In Sierra Leone, the revenue which was used to fund the civil conflict which destroyed much of the country’s health infrastructure is thought to have come from illegally traded diamonds [3]. This state of affairs raises significant moral and ethical concerns for consumers in high–income countries about the circumstances surrounding the production of their consumer goods. It also highlights the collective influence that can potentially be wielded by consumers to effect change through alterations in their purchasing habits. By engaging in so called ‘ethical consumerism’ and refusing to purchase goods whose production may have involved illegal resource expropriation, collective consumer action has the power to significantly reduce the resources available to armed groups and other perpetrators of serious human rights abuse [4].

## ETHICAL CONSUMERISM

Ethical consumerism involves the alteration of purchasing patterns to promote a certain set of moral values. Commonly, this involves boycotting products considered to be unethical and favoring the purchase of products which minimise harm to humans, animals or the environment. For example, ethical consumer movements such as the “Fair Trade Foundation” have presented consumers with an alternative range of food and clothing products whose sale results in a larger proportion of the purchasing price being allocated to producers in developing countries. This approach has been successful in utilizing market forces to improve trading standards, stimulate economic development and accelerate progress toward a number of Millennium Development Goal (MDG) targets. In terms of maternal health, quantitative impact data relating to Fair Trade interventions, has shown them to be beneficial both via the collateral effects of poverty reduction and through specific programs which have allowed women to earn a living wage and to reinvest some of their earnings in the development of health care infrastructure [6].

The principle of consumers and indeed nations having the right to base their purchasing patterns on ethical considerations is enshrined within international trade law [7,8]. A growing body of evidence demonstrates that the loss of revenue that results from ethically motivated collective consumer action, can act as a potent stimulant for behavior change among exploitative producers and intermediary companies [9]. The ability of consumers to make informed ethical choices is however confounded by the highly complex and convoluted nature of international trade relationships. The manufacturing, processing and distribution of many products frequently involve tangled chains of multiple, international, participants. Any participant can behave in ways that may be considered unethical, not only by the end–consumer, but also by the other participants in the trade chain. Providing complete transparency for consumers about the actions of all participants within a trade chain is therefore difficult, but not impossible. The illegal expropriation of natural resources in sub–Saharan Africa, most notably the DRC provides an illustrative case in point.

## MATERNAL MORTALITY AND CONFLICT MINERALS IN THE DEMOCRATIC REPUBLIC OF CONGO

The civil conflict in the Democratic Republic of Congo is estimated to have claimed upwards of 4 million lives since 1998 [10] and has been fuelled to a large extent by competition for the country’s natural resources. The mineral resources in the DRC have been estimated to have a value of 15 Trillion pounds – more than the combined GDP of Europe and the United States [11]. In spite of this vast potential wealth, in 2013 the DRC was placed 186th out of 187 countries in the UN Human Development Index, which ranks nations based on a composite statistic of life expectancy, education and indices of income inequality [12]. At the height of the civil conflict, estimates suggested the maternal mortality ratio in the DRC was upwards of 1100 per 100 000 [1]. The eastern DRC also suffers from the highest rates of sexual violence anywhere in the world with rape frequently being employed as a weapon of war. One study reported an incidence of 48 rapes occurring every hour in the eastern DRC [13]. At the height of the civil war between 1998 and 2003, an estimated 98% of the mining industry in DRC was driven by enforced labor under the control of various militia groups. The main objects of expropriation were gold and minerals such as Casserite (tin ore), Wolframite (tungsten ore) and Coltan (tantalum ore) which are essential for the manufacture of consumer electronics such as mobile phones. Investigations undertaken by an Expert Panel at the request of the UN Secretary General identified that these minerals are distributed globally to be used in manufacturing processes via multiple intermediary companies, several of which are UK–based [4]. Eventually, the minerals end up in the hands of consumers who through the purchase of phones, laptops and various other electronics, inadvertently helped to fund and perpetuate a conflict which has been described as “the world’s deadliest humanitarian crisis” [8].

**Figure:**
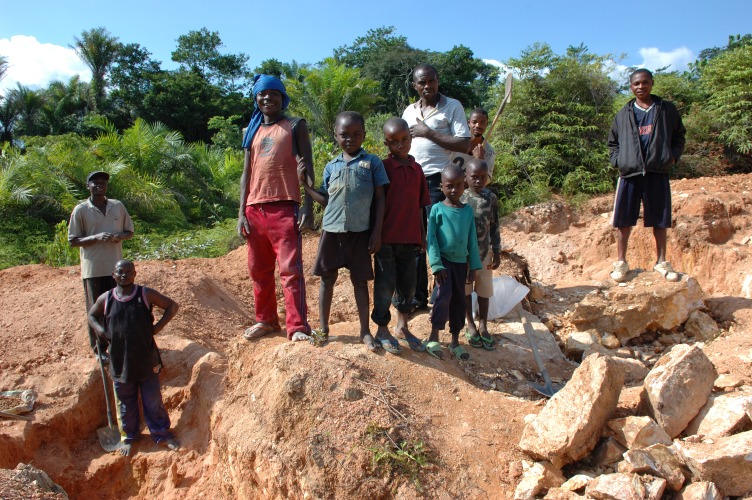
Photo: Child labor: artisan mining in Kailo, DR Congo. By Julien Harneis (Flickr: Mining in Kailo) [CC BY–SA 2.0 (http://creativecommons.org/licenses/by-sa/2.0)], via Wikimedia Commons.

In 2011, the United States senate passed a landmark reform on trade transparency in the form of the “Dodd–Frank Wall Street Reform and Consumer Protection Act”. As a result of campaigning from numerous lobby groups, Section 1502 of the act stipulates that companies operating in the US whose products contain Cassiserite, Wolframite and Coltan must submit to the Federal Securities and Exchange Commission, whether their minerals have been sourced in the DRC or surrounding countries. Companies who report that they do source their minerals from these regions must detail the measures that they have taken to ensure that they have not been obtained from armed groups that have been involved in massacres and other atrocities. The legislation is a demonstration of the power of collective consumer activism and lobbying, to effect legislative change at the highest level. The transparency that the act affords also provides consumers with the information required to make informed choices about their purchases. In 2014, the anti–genocide campaign group “The Enough Project” reported that most of the mineral mines in Congo were no longer under the control of militia groups. This transition has been lauded as a triumph of consumer activism with the changes being attributed to the Dodd–Frank Act and the ensuing efforts of electronics manufacturers to examine and reform their supply chains [9]. As a result of the marked reduction in the availability of financial capital to fuel ongoing conflict, increasing amounts resources are being diverted toward health care and other areas of societal development. With regards to maternal health, there is a renewed focus on the provision of skilled birth attendance, emergency obstetric care and improving access to contraception [14]. In 2015, the maternal mortality ratio in DRC was estimated to be around 693 per 100 000 [1]. This figure still represents one of the highest maternal mortality ratios in the world and is subject to a degree of uncertainty due to the challenges of accurately measuring mortality in regions without formal civil registration systems. Nevertheless, the data are highly suggestive of a marked reduction in maternal mortality since the end of the civil conflict.

## CONCLUSION

A maternal death, no matter where in the world it occurs, will be embedded in a complex network of biological, cultural, political and socio–economic causal factors. Sadly, vital branches of the networks that contribute to so many maternal deaths in sub–Saharan Africa extend across countries and continents to involve consumers from all over the globe. The World Health Organization has highlighted that a significant drawback of MDG 5 was the insufficient attention that it paid to broader development principles such as human rights and poverty reduction [15]. Ethical consumerism provides a means by which individuals and advocacy groups concerned with maternal mortality can actively engage with these issues. This approach, employed in conjunction with sanctions and international legal prosecutions has also been endorsed by the UN as a powerful means by which the international community can contribute toward creating an environment of sustainable, basic security in sub–Saharan Africa where improved health infrastructure can continue to develop and save mothers lives [4]. The need for our collective action becomes all the more apparent if we consider that in spite of the progress of the last quarter century, sub–Saharan Africa still has more than 160 000 maternal deaths every year, which is now more than half of the entire global burden [1].

A number of organisations collate data about company and country trade practices and present the information in the form of “ethical shopping guides” to help consumers make informed choices. These organisations include “The Ethical Consumer Organisation”, “The Ethical Company Organisation” and the “Fair Trade Foundation”. Other groups such as Raise Hope for Congo, The Enough Campaign and Fairphone deal directly with issues relating to conflict minerals in the DRC. The websites of any of these organisations are an ideal starting point for anyone wishing to engage with ethical consumerism.
